# High-Dose Fluoride Impairs the Properties of Human Embryonic Stem Cells via JNK Signaling

**DOI:** 10.1371/journal.pone.0148819

**Published:** 2016-02-09

**Authors:** Xin Fu, Fang-Nan Xie, Ping Dong, Qiu-Chen Li, Guang-Yan Yu, Ran Xiao

**Affiliations:** 1 Research Center of Plastic Surgery Hospital, Chinese Academy of Medical Sciences and Peking Union Medical College, 33 Ba Da Chu Road, Beijing, 100144, PR China; 2 Department of Oral and Maxillofacial Surgery, Peking University School of Stomatology, 22 Zhong Guan Cun South St., Beijing, 100081, PR China; Katholieke Universiteit Leuven, BELGIUM

## Abstract

Fluoride is a ubiquitous natural substance that is often used in dental products to prevent dental caries. The biphasic actions of fluoride imply that excessive systemic exposure to fluoride can cause harmful effects on embryonic development in both animal models and humans. However, insufficient information is available on the effects of fluoride on human embryonic stem cells (hESCs), which is a novel *in vitro* humanized model for analyzing the embryotoxicities of chemical compounds. Therefore, we investigated the effects of sodium fluoride (NaF) on the proliferation, differentiation and viability of H9 hESCs. For the first time, we showed that 1 mM NaF did not significantly affect the proliferation of hESCs but did disturb the gene expression patterns of hESCs during embryoid body (EB) differentiation. Higher doses of NaF (2 mM and above) markedly decreased the viability and proliferation of hESCs. The mode and underlying mechanism of high-dose NaF-induced cell death were further investigated by assessing the sub-cellular morphology, mitochondrial membrane potential (MMP), caspase activities, cellular reactive oxygen species (ROS) levels and activation of mitogen-activated protein kinases (MAPKs). High-dose NaF caused the death of hESCs via apoptosis in a caspase-mediated but ROS-independent pathway, coupled with an increase in the phospho-c-Jun N-terminal kinase (p-JNK) levels. Pretreatment with a p-JNK-specific inhibitor (SP600125) could effectively protect hESCs from NaF-induced cell death in a concentration- and time-dependent manner. These findings suggest that NaF might interfere with early human embryogenesis by disturbing the specification of the three germ layers as well as osteogenic lineage commitment and that high-dose NaF could cause apoptosis through a JNK-dependent pathway in hESCs.

## Introduction

Fluorides are inorganic and organic fluorine compounds that are widely used in numerous dental products for the prevention and remineralization of dental caries [1–2]. Low-dose fluorides are beneficial to bone health and have been used in the treatment of age-related osteoporosis for the last 40 years [3]. However, the biphasic actions of fluoride suggest that excessive systemic exposure to fluorides can lead to the disturbance of bone homeostasis (skeletal fluorosis) and enamel development (dental/enamel fluorosis) [4]. Similarly, acute and high-dose exposure to fluorides can result in renal toxicity [5], liver damage [6], neurological defects [7], reproductive toxicity [8], infertility [9] and mental retardation [10]. Moreover, high-dose fluoride can readily cross the placental barrier to directly damage the developing mammalian fetus, thus resulting in embryonic and fetal developmental abnormalities in a number of species, including frogs [11], rats [12] and mice [13]. Positive correlations between the fluoride content and pathological changes in the femur of aborted human fetuses have also been reported [14]. Numerous epidemiological and clinical studies have also demonstrated that high-dose fluorides could lead to changes in teeth and bone structure and adversely affect neurodevelopment by lowering the intelligence quotient (IQ) in children [10]. All of these findings suggested that high-dose fluoride could influence the development of the human embryo [11]. However, very little is known about the potential developmental toxicity and the underlying mechanism of high-dose fluorides on the early development of human embryos due to the lack of appropriate humanized models.

The successful *in vitro* culture of pluripotent human embryonic stem cells (hESCs) isolated from human blastocyst [15] created a new avenue to analyze the cytotoxicity and embryotoxicity of chemical compounds and substances in humans [16], as the *in vitro* differentiation of hESCs can partially recapitulate cellular developmental processes and gene expression patterns of early human embryogenesis [17]. For instance, formation of embryoid bodies (EBs), which are cell aggregates produced during the course of hESCs differentiation in suspension [18], indicating the onset of differentiation of hESCs during early embryogenesis [17]. Accumulating evidence also indicates that EB is spatially and temporally patterned [18] and the expressions of developmental marker genes can be used to define the EB morphogenesis [19–20]. Therefore, hESCs-based systems are currently being explored as alternatives for assessing the embryotoxic potential of compounds [21–23].

The effects of fluoride on cellular metabolism and physiology are diverse and are dependent upon the cell type, duration of exposure and concentration [24]. For instance, low-dose fluoride can exhibit a specific mitogenic effect on osteoblasts [25], enhance the osteoblastic differentiation of mesenchymal stem cells (MSC) [26], and induce the early differentiation of murine bone marrow cells along the granulocytic pathway [27]. In contrast, growth arrest and apoptosis induction are among the most common toxic effects of high fluoride levels on many types of cells, including ameloblasts [28], osteoblasts [29], epithelial cells [30] and mouse embryonic stem cells [31]. Elucidating the effects of high-dose fluorides on hESCs is thus important for understanding the impairment of early human embryogenesis.

In this study, we examined the differentiation, proliferation, viability and apoptosis of H9 hESCs under treatment with different concentrations of sodium fluoride (NaF). Furthermore, the mechanism of high-dose NaF on apoptosis in hESCs was investigated by assessing the mitochondrial membrane potential (MMP), caspase activities, cellular reactive oxygen species (ROS) levels and activation of mitogen-activated protein kinases (MAPKs). Our findings suggested that high-dose NaF suppressed proliferation and induced apoptosis in hESCs and disturbed the gene expression patterns of hESCs during EB differentiation. High-dose NaF-induced apoptosis in hESCs is facilitated by MAPK-mediated and caspase-dependent pathways and is independent of ROS production. To our knowledge, this is the first report to study the effects of high-dose fluorides on the biological characteristics of hESCs. Our findings may provide new insights into the mechanism of high-dose NaF-induced toxicities during early human embryogenesis.

## Materials and Methods

### Chemicals

Fluorescent probe JC-1 (5,5,6,6-tetrachloro-1,1,3,3-tetraethyl-imidacarbocyanine iodide) and inhibitors for pan-caspase (z-VAD-fmk), JNK (SP600125) and ERK (PD98059) were purchased from Beyotime (Jiangsu, China) and dissolved in dimethyl sulfoxide (DMSO). Unless otherwise specified, the chemicals used in this study were purchased from Sigma (St. Louis, MO, USA).

### Cell Culture

The H9 hESCs (WA09) was obtained from the WiCell Research Institute (Madison, WI, USA) under a Materials Transfer Agreement (No. 11-W0039). Cells were cultured on plates pre-coated with Matrigel (BD, Franklin Lakes, NJ, USA) and maintained in mTeSR-1 medium (StemCell Technologies, Vancouver, Canada) with daily medium change. When the cells reached 90% confluence, cells were scraped from the plate mechanically after dispase digestion (1 mg/ml, 2–3 min) and seeded on a new Matrigel-coated plate at a splitting ratio of 1:6.

### EB Formation

Upon reaching 90% confluence, hESCs were digested with 1 mg/ml dispase and scraped from the plate, followed by suspension culture in EB differentiation medium containing DMEM/F-12 (Invitrogen, Carlsbad, CA, USA), 20% knockout serum replacement (Invitrogen), 1 mM L-glutamine (Invitrogen), 1% non-essential amino acids (Invitrogen) and 0.1 mM β-mercaptoethanol. After 24 h, EB medium was supplemented with 0.5 or 1 mM of NaF, and the media were changed every other day. EB samples were collected on the 5th day (5D), 7th day (7D) and 14th day (14D) for further analysis. The circularities of 5D EB were measured from 50 randomly selected EBs that formed in each culture condition using Adobe Photoshop CS3, San Jose, CA, USA).

### Real-Time Polymerase Chain Reaction (PCR)

For cDNA synthesis, 500 ng of RNA was reverse transcribed using M-MLV reverse transcriptase (Life Technologies, Carlsbad, CA, USA) following the manufacturer's instructions. Real-time PCR was performed using the Fast SYBR Green Master Kit and LightCycler 480 system (Roche, Basel, Switzerland) according to the manufacturer’s instructions. The expression level of each gene was normalized to the expression of *GAPDH*. Primer sequences were summarized in Table 1.

**Table 1:** List of primers used in the real-time PCR.

Genes	Primer sequences
*4-Oct*	F: 5’-CCCCTGGTGCCGTGAA-3’
	R: 5’-GCAAATTGCTCGAGTTCTTTCTG-3’
*Nanog*	F: 5’-ATGCCTCACACGGAGACTGT-3’
	R: 5’-AAGTGGGTTGTTTGCCTTTG-3’
*SOX2*	F: 5'-TTGCTGCCTCTTTAAGACTAGGA-3’
	R: 5'-CTGGGGCTCAAACTTCTCTC-3’
*NeuroD1*	F: 5’-CGCTGGAGCCCTTCTTTG-3’
	R: 5’-GCGGACGGTTCGTGTTTG-3’
*Brachyury*	F: 5’-CCTCYCCCYCCCCYCCACGC-3’
	R: 5’-GGTGGGCTGGCATTGTGGCT-3’
*AFP*	F: 5’-TGCAGCCAAAGTGAAGAGGGAAGA-3’
	R: 5’-CATAGCGAGCAGCCCAAAGAAGAA-3’
*NOS*	F: 5’-CACTCAGCTGTGCATCGAC-3’
	R: 5’-CAGTTCCCGAAACCACTCGT-3’
*SOD2*	F: 5’-AACAACCTGAACGTCACCGA-3’
	R: 5’-AGCAACTCCCCTTTGGGTTC-3’
*CYBA*	F: 5’-GAGCGGCATCTACCTACTGG-3’
	R: 5’-TGATGGTGCCTCCGATCT-3’
*PRDX5*	F: 5’-GTTCGGCTCCTGGCTGATCC-3’
	R: 5’-TGCCATCCTGTACCACCAT-3’
*RUNX2*	F: 5’-ACTGGCGCTGCAACAAGAC-3’
	R: 5’-CCCGCCATGACAGTAACCA-3’
*OPN*	F: 5’-CAGCAACCGAAGTTTTCACTCCAG-3’
	R: 5’-CACCATTCAACTCCTCGCTTTCC-3’
*COL1A*	F: 5’-GTGAACCTGGTGCTCCTG-3’
	R: 5’-GTGAACCTGGTGCTCCTG-3’
*PPAR-*γ	F: 5’-TGGAATTAGATGACAGCGACTTGG-3’
	R: 5’-CTGGAGCAGCTTGGCAAACA-3’
*CEBP/α*	F: 5’-AGGAACACGAAGCACGATCA-3’
	R: 5’-ACAGAGGCCAGATACAAGTG-3’
*Adiponectin*	F: 5’-CTTCCGTCACCTCTAAATCC-3’
	R: 5’-GTCATCCCTAACTTCAGTGG-3’
*GAPDH*	F: 5’-GCACCGTCAAGGCTGAGAAC-3’
	R: 5’-TGGTGAAGACGCCAGTGGA-3

The oligonucleotide sequences of primers used in the real-time PCR analysis

### Cell Proliferation and Viability Assay

The hESCs (5×10^3^ cells/well) were plated into 96-well plates containing 100 μl of mTeSR-1 medium and exposed to increasing concentrations of NaF (1~6 mM) at 37°C for 24 to 120 h in the presence or absence of pharmacological inhibitors. The number of viable cells was quantified by the CellTiter 96 AQueous One Solution Cell Proliferation kit (Promega, Wisconsin, USA) following the manufacturer’s instructions. In brief, 20μl of (3-(4,5-dimethylthiazol-2-yl)-5-(3-carboxymethoxyphenyl)-2-(4-sulfophenyl)-2H-tetrazolium (MTS) was added in each well and incubated for 4 h at 37°C. The absorbance was measured at 490 nm on a PerkinElmer EnSpire^TM^ Multimode Plate Reader. All experiments were repeated three times with triplicates in each experiment.

### Cell Apoptosis Analysis by Annexin V/propidium iodide (PI) Staining

The hESCs exposed to NaF were incubated with the Muse Annexin V Dead Cell Kit (EMD Millipore, Billerica, MA, USA) following the manufacturer’s instructions. The percentages of apoptotic cells were analyzed with a program for Annexin V and dead cells on the Muse Cell Analyzer (EMD Millipore).

### Transmission Electron Microscopy (TEM)

Alternations in the ultramicrostructure of the hESCs after NaF exposure were observed by TEM. In brief, the cells were dissociated into single cells by accutase (Stemcell Technologies) treatment and centrifuged to remove supernatant. The cell pellets were then fixed with 2.5% glutaraldehyde overnight at 4°C, followed by fixation, staining, dehydration, embedding and sectioning as described previously [32]. The ultrathin sections were stained with uranyl acetate and lead citrate and then observed by TEM (H-7650, Hitachi, Japan) at 80 kV.

### Measurement of MMP and Caspase Activity

The hESCs (1×10^4^ cells/well) were seeded into 96-well plates and cultured in mTeSR-1 medium for 4 days. On day 5, hESCs were exposed to increasing concentrations of NaF (0.5, 1, 2 and 4 mM) for 24 h. To run the JC-1 assay, cells were rinsed with PBS once and then incubated with JC-1 working solution (mTeSR-1 medium supplemented with 10 μg/ml JC-1) at 37°C for 1 h, followed by two washes with PBS. The mTeSR-1 medium was then added back to the plate, and the fluorescence intensities of the cells were monitored at Ex/Em = 490/525 nm (green) and 490/590 (red) nm on a Multimode Plate Reader. The ratio of red-to-green fluorescence was calculated, and the loss of MMP was indicated by the decrease in the ratio. The caspase activities of the cells were assessed by the Caspase-Glo^®^ 3/7 Assay System (Promega, Wisconsin, USA) in the absence or presence of 20 μM z-VAD-fmk.

### Measurement of Intracellular ROS

A stock solution of 2,7-dichlorodihydrofluorescein-diacetate (DCFH-DA, 25 mM) was prepared in DMSO and stored at -20°C in the dark. The hESCs exposed to increasing concentrations of NaF (1~4 mM) were incubated with DCFH-DA (5 μM) for 10 min. The single cell suspension was prepared after accutase treatment and 1×10^4^ events/cell sample were recorded by FACSAria™ II (BD). The percentage of green-fluorescence positive cells and the mean value of green fluorescence intensities were calculated using the Flowjo v7.6.1 software (Tree Star, OR, USA).

### Western Blot Analysis

After NaF treatment, total protein from hESCs was isolated using the mirVana™ miRNA Isolation kit (Life technologies). Protein lysates (50 μg/sample) were analyzed by western blot using primary antibodies, including mouse anti-human JNK (SC-7345, Santa Cruz Biotechnology, Santa Cruz, CA), mouse anti-human p-JNK (SC-293136, Santa Cruz Biotechnology), rabbit anti-human ERK (GTX59618, Genetex, Irvine, CA), rabbit anti-human p-ERK (2219–1, Epitomics, Burlingame, CA), rabbit anti-human BCL-2 (GTX100064, Genetex), rabbit anti-human BAX (GTX109683, Genetex) and mouse anti-human GAPDH (TA08, ZSGB-BIO, Beijing, China). The horseradish peroxidase (HRP)–conjugated donkey anti-mouse IgG (ZB5305, ZSGB-BIO) or donkey anti-rabbit IgG (ZB5301, ZSGB-BIO) were used as secondary antibodies. SuperSignal West Pico Trial Kit (Thermo Scientific, Rockford, IL) was applied for protein detection. The intensity of individual bands was quantified using the ImageJ densitometry software.

### Statistical Analysis

Statistical analysis was performed using the SPSS statistical software package. Differences were evaluated by an independent Student’s t-test and paired t-test. A *p*-value < 0.05 was considered statistically significant.

## Results

### NaF Affected the Gene Expression Patterns of hESCs during EB Differentiation

**Fig 1:**
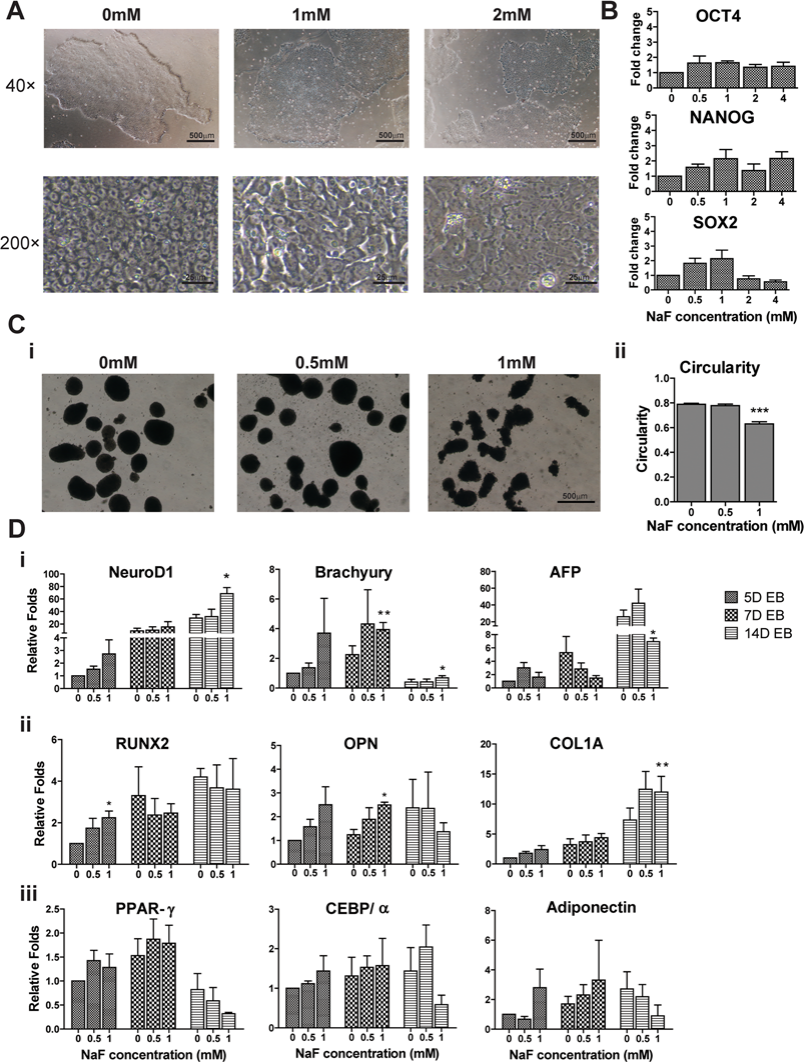
Sodium Fluorides (NaF) affected the differentiation of H9 hESCs. (A) The morphology of hESCs was characterized under an inverted microscope. NaF-treated hESCs were larger and flattened than untreated cells were. (B) The expressions of pluripotent markers (*OCT4*, *NANOG* and *SOX2*) in untreated and NaF-treated hESCs were comparable, as quantified by real-time polymerase chain reaction (PCR). (C) The morphology of embryoid bodies (EBs) derived from hESCs after 5 days (5D) of differentiation in the presence or absence of NaF. (i) 5D EBs in untreated and 0.5 mM NaF-treated groups exhibited a circular in shape while EBs in the 1 mM NaF-treated group were irregular in shape. (ii) The circularities of 5D EBs in the 1 mM NaF group were significantly lower than in the untreated and 0.5 mM NaF-treated groups. (D) The gene expression patterns of EBs were disturbed by high-dose NaF treatment. (i) The expression of three germ layer markers (Ectoderm: *NeuroD1*, Mesoderm: *Brachyury*, Endoderm: *AFP*) in EBs. (ii) Expression of osteogenesis markers (*RUNX2*, *OPN* and *COL1A*) in EBs. (iii) Expression of adipogenesis markers (*PPAR-γ*, *CEBP/α*, *Adiponectin*) in EBs. 40×: 40× magnification. 200×: 200× magnification. *, *p* < 0.05. **, *p* < 0.01. ***, *p* < 0.001.

This study was the first to examine the effects of NaF on the biological characteristics of H9 hESCs, including the morphology, expression of pluripotent markers (*OCT4*, *NANOG* and *SOX2)* and differentiation potential by inducing the differentiation of hESCs into EB. Observations at 40× magnification showed that H9 hESCs grew as compact colonies with distinct borders in both the untreated (0 mM) and NaF-treated (1 mM, 2 mM) groups; in contrast, at higher magnification (200×), the untreated hESCs exhibited typical hESC morphology (small and tightly packed with prominent nuclei), but the NaF-treated hESCs became larger and flattened (Fig 1A). The expression of pluripotent markers (*OCT4*, *NANOG* and *SOX2*) in hESCs was not significantly affected after NaF treatment (Fig 1B). After 5 days of hESC differentiation, the EBs exhibited a circular shape in both untreated and 0.5 mM NaF-treated culture but appeared irregular in the 1 mM NaF-treated culture, with a significant decrease in circularity (***, *p* < 0.001, Fig 1C). Moreover, 1 mM NaF significantly up-regulated the expressions of the ectoderm marker *NeuroD1* in 14D EB and the mesoderm marker *Brachyury* in 7D and 14D EB but markedly decreased the expression of the endoderm marker *AFP* in 14D EB (Fig 1Di). Notably, 1 mM NaF could also disturb the osteogenesis of the EBs by significantly up-regulating the osteogenesis markers *RUNX2*, *OPN* and *COL1A* at different time points (*, *p* < 0.05, Fig 1Dii). However, no significant impact of NaF on the expression of adipogenesis markers (*PPAR-γ*, *CEBP/α*, *Adiponectin*) in EBs was detected (Fig 1Diii).

### High-Dose NaF Affected the Proliferation and Viability of hESCs

**Fig 2:**
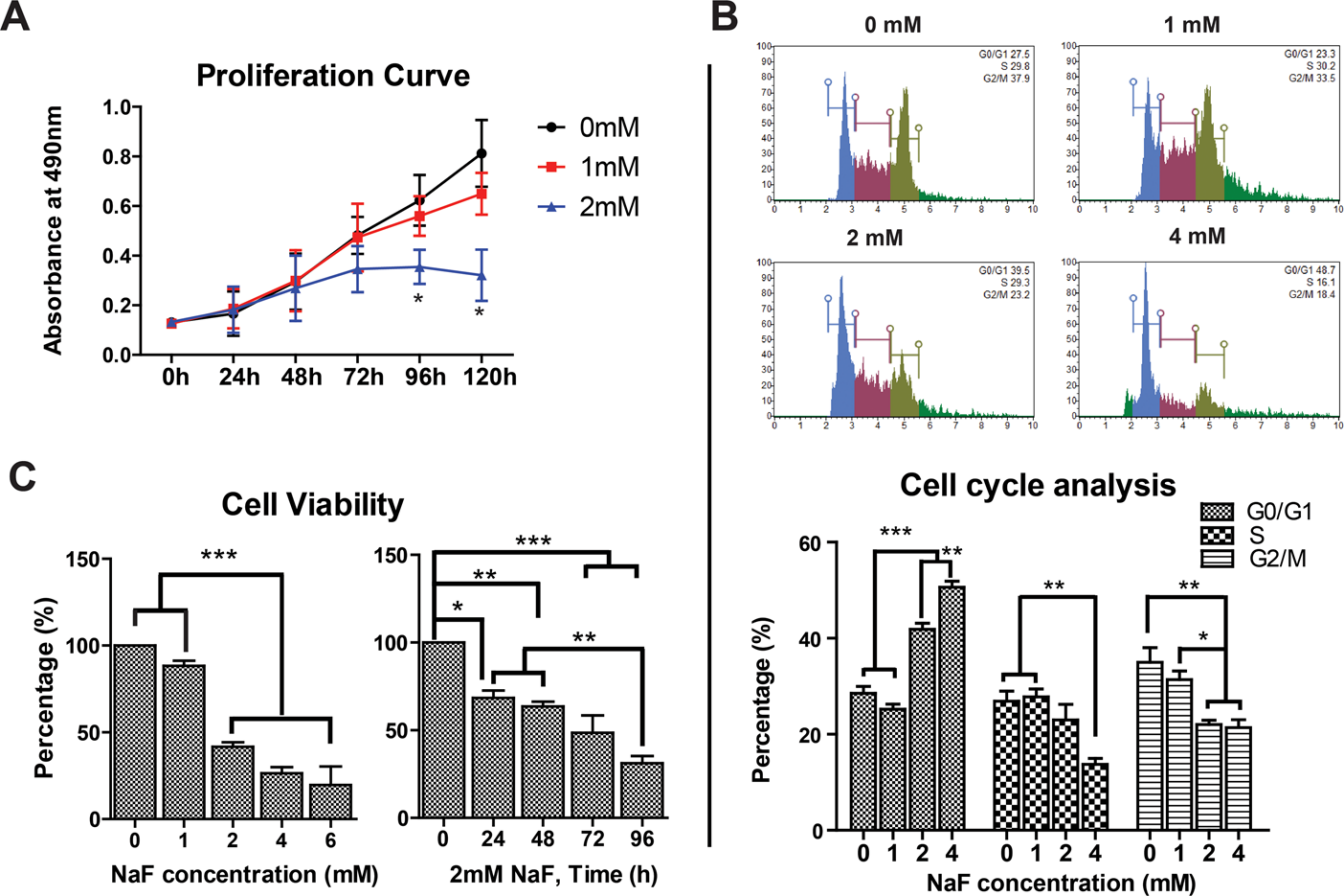
High-dose NaF impaired the proliferation and viability of hESCs. (A) Proliferation of hESCs in the 2 mM NaF-treated group was significantly lower than in the untreated and 1 mM NaF-treated groups. (B) Cell cycle arrest in G0/G1 phase was induced in H9 hESCs after exposure to 2 and 4 mM NaF. (C) Cell viability of hESCs was dramatically decreased in the 2, 4 and 6 mM NaF-treated groups, with a time-dependent reduction in the 2 mM NaF-treated group. *, *p* < 0.05. **, *p* < 0.01. ***, *p* < 0.001.

To assess the influence of high-dose NaF on hESC proliferation, the number of viable cells was determined by measuring the absorbance at 490 nm using an MTS assay during the experimental periods. A time-dependent increase in the absorbance was observed in both the untreated and 1 mM NaF-treated groups, but in the 2 mM NaF-treated group, the proliferation of hESCs was markedly suppressed after 96 h of culture (Fig 2A, *, *p* < 0.05). Flow cytometry analysis after PI staining showed that 2 and 4 mM NaF could induce cell cycle arrest in hESCs, thus leading to a significant increase in the number of cells in G0/G1 phase with a concomitant decrease of cells in S and G2/M phase (Fig 2B, *, *p* < 0.05, **, *p* < 0.01, ***, *p* < 0.001). Consistently, the viability of hESCs was dramatically decreased in the 2, 4 and 6 mM NaF-treated groups, with a time-dependent reduction in the 2 mM NaF-treated group (Fig 2C, *, *p* < 0.05, **, *p* < 0.01, ***, *p* < 0.001).

### High-Dose NaF Induced Apoptosis in hESCs

**Fig 3:**
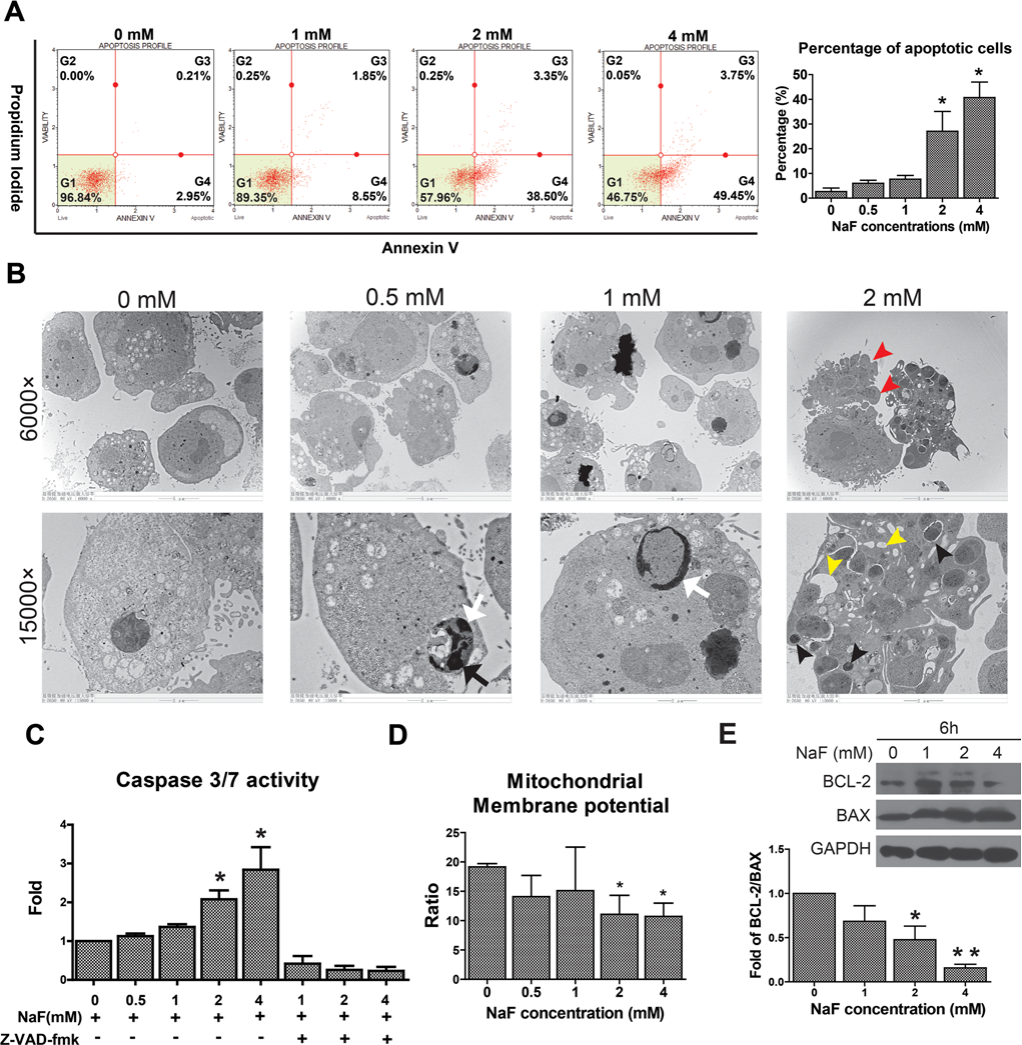
High-dose NaF induced the occurrence of apoptosis in H9 hESCs. (A) The percentage of early apoptotic cells (G3) and late apoptotic cells (G4) was significantly increased after exposure to 2 mM and 4 mM NaF, as demonstrated by the Annexin V/ Propidium Iodide staining assay. (B) The sub-cellular morphology of hESCs was characterized by transmission electron microscopy, and the data showed that NaF-treated hESCs exhibited morphological and nuclear features typical of apoptosis, including nuclear fragmentation (black arrow), chromatin condensation (white arrow), shrinkage and blebbing of the plasma membrane (red arrowhead), cytoplasmic vacuoles (yellow arrowhead) and the formation of apoptotic bodies (black arrowhead). (C) Caspase activities were significantly increased, whereas the (D) mitochondrial membrane potential (MMP) and the (E) BCL-2/BAX ratio were significantly decreased in hESCs after exposure to 2 and 4 mM NaF for 24 h. *, *p* < 0.05. **, *p* < 0.01.

Because a reduction in cell viability indicates the occurrence of cell death, NaF-treated hESCs were analyzed by flow cytometry after Annexin V/PI staining. The percentage of cells gated in G4 (early apoptotic cells) and G3 (late apoptotic cells) increased after exposure to NaF (Fig 3A). The TEM results confirmed that the untreated hESCs exhibited normal morphologies with an intact plasma membrane and clear nucleus. In contrast, the morphological characteristics of the early phases of apoptosis, including nuclear fragmentation (black arrow) and chromatin condensation (white arrow), were observed in 0.5 and 1 mM NaF-treated hESCs (Fig 3B). Additionally, 2 mM NaF-treated hESCs showed characteristics of late stage of apoptosis, including shrinkage and blebbing of the plasma membrane (red arrowhead), cytoplasmic vacuoles (yellow arrowhead) and the formation of apoptotic bodies (black arrowhead).

The high-dose NaF-induced apoptosis was further verified by measuring the activities of caspase3/7, the cellular levels of MMP and the expression of apoptosis regulators (BCL-2 and Bax) in hESCs. The caspase3/7 activity was significantly increased in 2 and 4 mM NaF-treated hESCs, which could be inhibited by the pan-caspase inhibitor z-VAD-fmk (Fig 3C). In these two NaF-treated groups, mitochondrial function analysis also revealed a significant decrease in both the cellular MMP levels and the Bcl-2/BAX ratio in hESCs (Fig 3D).

### High-Dose NaF-Induced Apoptosis in hESCs Was ROS-Independent

**Fig 4:**
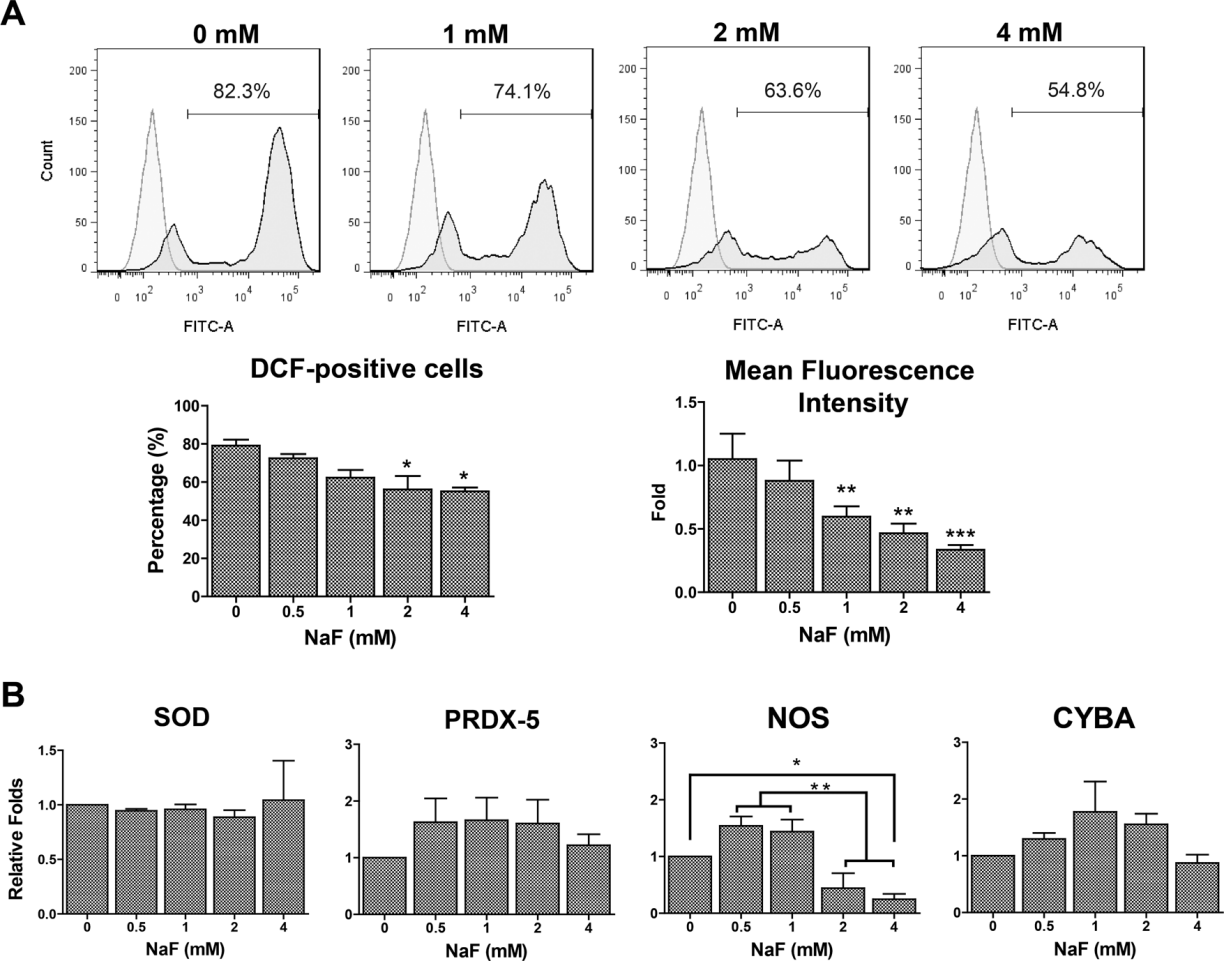
Production of intracellular reactive oxygen species (ROS) in hESCs was decreased after NaF treatment. (A) Assessment of ROS levels by 2,7-dichlorodihydrofluorescein-diacetate (DCFH-DA) staining revealed that the percentage of fluorescent dichlorofluorescein (DCF)-positive hESCs was significantly decreased in the 2 and 4 mM NaF-treated groups, and the mean fluorescence intensities of hESCs were significantly lower in the 1, 2 and 4 mM NaF-treated groups. (B) The expression of the cellular antioxidant defense enzyme *SOD* and the oxidative stress genes *PRDX5* and *CYBA* did not show significant changes after NaF treatment, except for *NOS*, whose expression was significantly decreased in the 2 and 4 mM NaF-treated hESCs. *, *p* < 0.05. **, *p* < 0.01. ***, *p* < 0.001.

Because the involvement of increased ROS production has been proven in NaF-induced apoptosis in some cell types, the intracellular ROS levels in NaF-treated hESCs were investigated by DCFH-DA staining followed by flow cytometry analysis. The representative flow cytometry histograms showed decreased ROS levels in NaF-treated hESCs in a dose-dependent manner (Fig 4A). Three independent experiments revealed that the percentages of dichlorofluorescein (DCF)-positive cells were significantly decreased in 2 and 4 mM NaF-treated hESCs and that the mean fluorescence intensities of hESCs were significantly lower in the 1, 2 and 4 mM NaF groups than in the untreated group. The expression of the cellular antioxidant defense enzyme *SOD* and the oxidative stress genes *PRDX5*, *NOS* and *CYBA* were further characterized by real-time PCR, and the results showed that NaF treatment did not alter the expression levels of *SOD*, *PRDX5* and *CYBA* in hESCs, except for *NOS*, whose expression was significantly decreased in the 2 and 4 mM NaF-treated hESCs (Fig 4B).

### JNK-Mediated Signaling Was Involved in High-Dose NaF-Induced Apoptosis in hESCs

**Fig 5:**
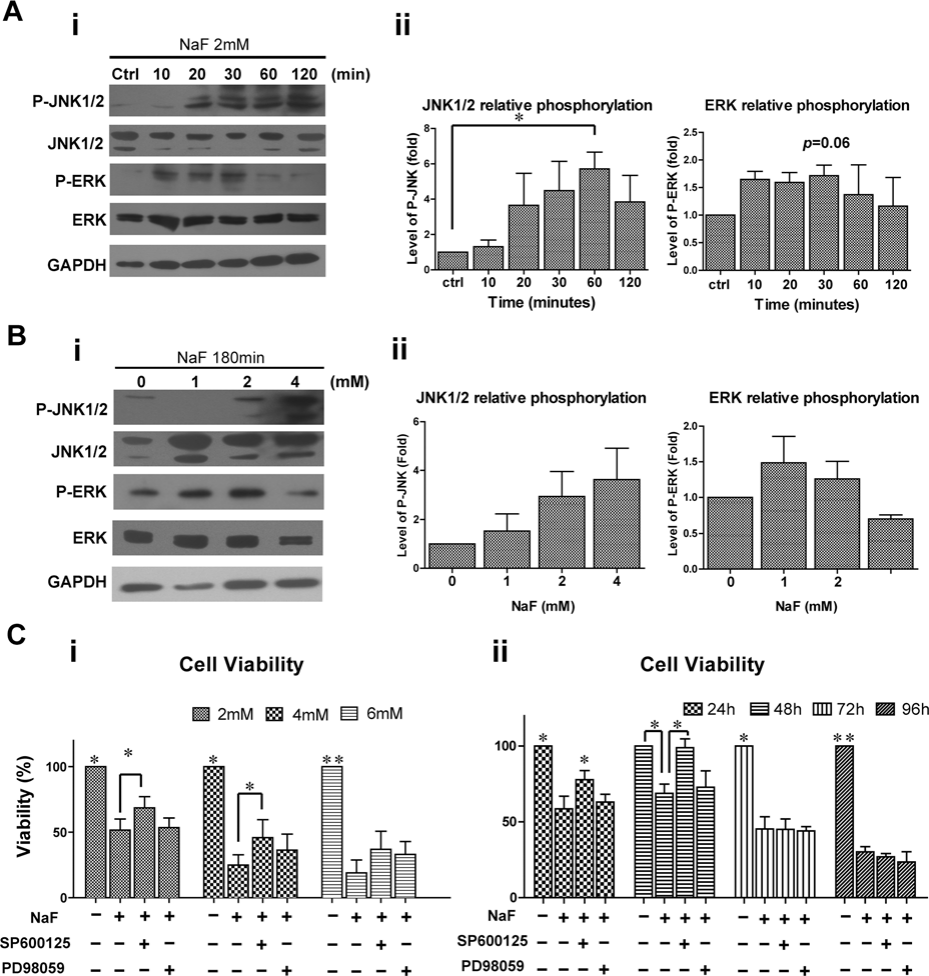
High-dose NaF induced apoptosis in hESCs via the mitogen-activated protein kinase (MAPK)-dependent pathway. (A) Time-dependent effects of 2 mM NaF on the activation of MAPKs in hESCs. (i) Western blot analysis showed a time-dependent increase in the levels of phosphorylated-c-Jun N-terminal kinases 1/2 (P-JNK1/2) and a transient increase in the levels of phosphorylated-extracellular signal-regulated kinases (P-ERKs) in 2 mM NaF-treated hESCs. (ii) Three independent experiments demonstrated that the levels of P-JNK1/2, but not P-ERK, were significantly increased after 60 min of NaF treatment. (B) Dose-dependent effects of NaF on the activation of MAPKs in hESCs. (i) Western blot analysis showed a dose-dependent increase in the levels of P-JNK1/2 and a transient increase in the levels of P-ERK in hESCs treated with increasing doses of NaF for 180 min. (ii) Three independent experiments demonstrated no significant difference in the levels of P-JNK1/2 and P-ERK between untreated and NaF-treated hESCs. (C) Verification of the effects of NaF-induced phosphorylation of JNK and ERK on the apoptosis of hESCs. (i) The viabilities of the 2 and 4 mM NaF-treated hESCs were significantly increased by pre-treating hESCs with the P-JNK1/2 specific inhibitor SP600125 but not with the P-ERK specific inhibitor PD98059. (ii) SP600125 but not PD98059 could significantly suppress the decreased cell viability of hESCs exposed to 2 mM NaF for 24 h and 48 h. *, *p* < 0.05. **, *p* < 0.01.

To investigate the roles of MAPKs in high-dose NaF-induced apoptosis, the activation of MAPK family members, extracellular signal-regulated kinases (ERKs) and c-Jun N-terminal kinases (JNKs) were examined in NaF-treated hESCs by western blot analysis. The results showed that the levels of phosphorylated-JNK1/2 (P-JNK1/2) were notably increased in NaF-treated cells in a time- (Fig 5A) and dose-dependent manner (Fig 5B). P-JNK1/2 levels in 2 mM NaF-treated hESCs reached a peak after 60 min of treatment (Fig 5Aii, *, *p* < 0.05). The levels of phosphorylated-ERK (P-ERK) in hESCs were only mildly increased after exposure to 2 mM NaF for 10 min to 30 min or exposure to 1 mM NaF for 180 min, but no significant difference was observed between untreated and NaF-treated hESCs (Fig 5Aii and 5Bii).

The effects of NaF-induced phosphorylation of JNK1/2 and ERK on apoptotic cell death were further verified by pre-treating H9 hESCs with the P-JNK1/2 inhibitor SP600125 (10 μM) or the P-ERK inhibitor PD98059 (20 μM) for 1 h before NaF treatment. The viabilities of the 2 and 4 mM NaF-treated hESCs were significantly increased by pre-treating hESCs with SP600125 (Fig 5C, *, *p* < 0.05), but not with PD98059. Neither SP600125 nor PD98059 could effectively prevent the cell death of hESCs induced by 6 mM NaF. Moreover, SP600125 could suppress the decreased cell viability of hESCs exposed to 2 mM NaF for 24 h and 48 h, but not for 96 h and 120 h (Fig 5Cii, *, *p* < 0.05). In contrast, PD98059 did not attenuate the high-dose NaF-induced cell viability decrease in hESCs by a significant level (Fig 5Cii).

## Discussion

It has been reported that hESCs can recapitulate both cellular developmental processes and gene expression patterns of early embryogenesis during *in vitro* differentiation [33]. Therefore, hESC is considered as an *in vitro* model to investigate the molecular mechanisms of embryonic cell differentiation [34–36] and embryotoxicity of developmental toxicants [37]. Previous studies have shown that ESCs require the expression of various transcription factors such as OCT4, NANOG and SOX2 to specify the stem cell state [38–39]. In addition, EB formation recapitulates features of pregastulation and early gastrulation [40–41] and the embryonic differentiation event is tightly regulated by the actions of specific gene products [20]. Therefore, expression of pluripotent and differentiation-related marker genes have been detected by transcriptome analysis to assessed the chemical impact on cell differentiation more sensitively, quantitatively, objectively, and speedily [20–21,23,42–43].

Fluoride is a ubiquitous natural substance that is present in the environment and is often used as an effective prophylactic for dental caries. However, the excess intake of fluorides impairs metabolism and cellular functions in different tissues and organs and ultimately leads to cell death [24]. Although harmful effects of high-dose fluorides on embryonic development have been reported in animal models [11–13] and in the developing brain of human fetuses [14], little information is available about the effects of fluorides on early human embryogenesis. In our study, for the first time, we showed that 1 mM NaF did not significantly affect the proliferation of hESCs but did disturb gene expression patterns during EB differentiation by suppressing the expression of endoderm markers while enhancing the expression of ectoderm, mesoderm and osteogenesis markers. Higher doses of NaF (2 mM and above) markedly decreased the viability and proliferation potential of hESCs and led to apoptosis via a ROS-independent and JNK-mediated pathway.

Our findings suggest that high-dose NaF might interfere with early human embryogenesis by disturbing both the regulation of the specification of the three germ layers and osteogenic lineage commitment. We observed a marked increase in *NEUROD1* expression in 14D EB after 1 mM NaF treatment. NEUROD1 is a basic helix-loop-helix transcription factor that has been identified as a differentiation factor for neurogenesis [44] and was found to strikingly coincide with terminal neural differentiation; further, its over-expression could lead to the premature and ectopic neural differentiation of neural stem cells both *in vitro* and *in vivo* [45]. Adverse effects of fluorides on neurodevelopment, such as DNA damage in cultured neural cells [7], behavioral deficits in rats [46] and lowered IQ in children [10], have been reported by numerous research groups. NaF-induced over-expression of *NEUROD1* in hESCs-differentiated EB suggested that the toxicity of high-dose fluorides on human neurodevelopment might be partially attributed to the ectopic and premature differentiation of neuronal precursors during early embryogenesis. In addition, NaF is a potent stimulator of ongoing osteogenesis from already differentiated osteoblasts, and it can substitute for a normal bone inducer and permit the initiation of osteoblastic differentiation of MSCs [26] as well as osteogenesis from the embryonic mesenchyme [47–48]. Consistently, our findings showed that 1 mM NaF increased the expression of *RUNX2* followed by the up-regulation of *OCN* and *COL1A* during EB differentiation, thus indicating that NaF could accelerate the osteogenic differentiation of hESCs.

Apoptosis, which is also known as programmed cell death, is a complex and highly regulated phenomenon that is characterized by a series of cellular processes, including chromatin condensation, DNA fragmentation, mitochondria disintegration, cell shrinkage, membrane blebbing and apoptotic body formation [24]. In our study, TEM analysis showed that high-dose NaF-treated hESCs exhibited morphological and nuclear features typical of apoptosis. Mitochondria are active participants in apoptosis and play central roles in both caspase-dependent and caspase-independent death pathways [49–50]. An important mitochondrial event during apoptosis is the reduction of MMP, which is accompanied by the alteration of Bcl-2 family proteins [31, 51]. The Bcl-2 family proteins, whose members may be anti-apoptotic or pro-apoptotic, regulate apoptosis by controlling mitochondrial permeability [52]. The down-regulation of anti-apoptotic proteins such as BCL-2 and the up-regulation of pro-apoptotic proteins such as BAX could cause MMP loss, thus allowing the release of cytochrome c out of the mitochondria and ultimately lead to the activation of caspase3/7, which act as executioners to initiate apoptotic cell death [53]. Our data demonstrated the reduction of MMP and the BCL-2/BAX ratio coupled with caspase 3/7 activation in high-dose NaF-treated hESCs and indicated that NaF induced apoptosis in hESCs occurs via a mitochondria-mediated and caspase-dependent pathway.

The mechanisms of fluoride-mediated apoptosis are under extensive study but are still not fully understood due to the complexity and diversity of the molecular events underlying cell–fluoride interactions [54]. Accumulating evidence has suggested that the increased production of cellular ROS is closely involved in high-dose fluoride-induced apoptosis in many cell types [24, 31, 54]. ROS are free radicals and chemically reactive molecules that contain oxygen, are normally generated during mitochondrial oxidative metabolism and induce oxidative stress in cells [55–56]. However, a lack of ROS production during high-dose fluoride-induced apoptosis has also been reported in several cell types [57–58]. Moreover, oxidative stress was absent in patients with skeletal fluorosis [59]. Therefore, the roles of ROS in high-dose fluoride-induced apoptosis might be cell-type dependent. It has been reported that stem cells possess low levels of intracellular ROS because they engage scavenger antioxidant enzyme systems [60]. Our finding of a reduced rather than increased production of ROS in NaF-treated hESCs might be partially explained by the robust expression of the antioxidant enzyme *SOD* and the decreased expression of the stress response gene *NOS* in hESCs.

MAPKs are a group of protein serine/threonine kinases that play important roles in complex cellular programs such as proliferation, differentiation and apoptosis [61]. Three subfamilies of MAPKs, including ERKs, JNKs and p38-MAPKs, have been identified, and ERKs have been shown to be important for cell survival, whereas JNKs and p38-MAPKs were deemed to be stress responsive and thus involved in apoptosis [62]. Indeed, a few scientific studies have demonstrated that activation of MAPKs was associated with fluoride-induced apoptosis [30–31]. In parallel with these reports, our results demonstrated a direct correlation between high-dose NaF-induced apoptotic cell death and JNK-activation in hESCs, thus confirming the involvement of JNK signaling. However, pretreatment with SP600125 could not effectively recover the decreased cell viability of hESCs exposed to 6 mM NaF for 24 h or 2 mM NaF for longer than 48 h. These findings suggested that other signaling pathways might be associated with the NaF-mediated cell death of hESCs undergoing acute exposure to NaF at concentrations higher than 6 mM or chronic exposure to 2 mM NaF for more than 2 days.

## Conclusions

In summary, our findings demonstrated that treatment with 1 mM NaF could disturb the gene expression patterns of hESCs during EB differentiation but that high-dose NaF (greater than 2 mM) caused suppressed proliferation and apoptosis in hESCs. Our data further revealed that JNK signaling was involved in NaF-induced hESC apoptosis in a concentration- and time-dependent manner. These findings suggested that chronic exposure to NaF over a threshold concentration might interfere with early human embryogenesis by disturbing the regulation of the specification of the three germ layers, impairing osteogenic lineage commitment, and causing apoptotic cell death in stem cells. Nevertheless, upstream factors that connect NaF treatment with MAPK activation were not discussed in our study, and further investigations are needed to expand on our findings.

## Abbreviations

hESCs: human embryonic stem cells
MSC: mesenchymal stem cell
EB: embryoid body
NaF: sodium fluoride
RT-PCR: real-time polymerase chain reaction
ROS: reactive oxygen species
MMP: mitochondrial membrane potential
